# Designing for climate change: twenty-five design features to improve sanitation technology resilience in low- and middle- income countries

**DOI:** 10.1007/s11027-024-10177-7

**Published:** 2024-11-13

**Authors:** Ian Cunningham, Jeremy Kohlitz, Juliet Willetts

**Affiliations:** Small is Beautiful Consulting, Australia; and Institute for Sustainable Futures, University of Technology Sydney, Ultimo, Australia

**Keywords:** Sanitation, Wastewater, Design, Climate change, Floods, Resilience, Risk, Sustainable, Decentralised

## Abstract

Climate change is exacerbating events such as floods and droughts, and trends including sea-level rise, leading to failures in sanitation technologies, increased public health risks and environmental pollution. To reduce these risks, it is crucial to incorporate climate resilience into sanitation technology designs. In this study, we reviewed academic and selected grey literature and identified 25 design features that can contribute to the technology’s resilience to an increasingly volatile and extreme climate. Design features that were conceptually similar were collated into seven categories. These categories included: (i) avoid exposure to hazards, (ii) withstand exposure to hazards, (iii) enable flexibility, (iv) contain failures, (v) limit consequences of complete failure, (vi) facilitate fast recovery and (vii) features that provide resilience benefits beyond technological resilience. In this paper we define the categories and design features, and provide examples of each feature in practice. We also outline how the resilience design features can support sanitation designers and implementers to critique the climate resilience of sanitation technology, and prompt more resilient designs of sanitation technology.

**Supplementary Information:**

The online version contains supplementary material available at 10.1007/s11027-024-10177-7.

## Introduction

Sanitation technologies in low- and middle-income countries (LMIC) are vulnerable to the effects of climate change. Failure of sanitation technologies can lead to public health risks and environmental pollution through the release of faecal pathogens, nutrients and other pollutants into the environment (Mills et al. 2020). Globally, 3.6 billion people, including 3.3 billion from LMICs, do not have access to safe sanitation (World Health Organization & UNICEF, 2021). More frequent and intense weather events such as floods, droughts, and storms, and changes in precipitation patterns and rising temperatures (Ranasinghe et al., 2021) place further stress on sanitation technologies, which is likely to then exacerbate poor access to sanitation.

Climate hazards can disrupt sanitation technology (Hyde-Smith et al. 2022). For example, sanitation structures, such as pits, containment units and treatment facilities, can be damaged or destroyed by the forceful impact of storm winds, floodwater, and the intensity of wildfires (Flood and Cahoon 2011; International Federation of Red Cross and Red Crescent Societies 2020). The flow or conveyance of waste through a sanitation technology can overflow or block because of extreme or variable precipitation (Botturi et al. 2021; Draude et al. 2020). Treatment processes may be disrupted by saltwater intrusion or changes in temperature (S. Singh and Tiwari 2019). Finally, inputs that are needed for sanitation technologies to function, such as water, electricity, or access for operation and maintenance, can be cut off by extreme weather events (Purwar et al. 2020).

Sanitation technologies that safely contain or treat human waste, despite climate hazards, are just one component of climate resilient sanitation services. A resilient sanitation service needs to consider the sanitation system. This includes non-infrastructure elements such as good governance and leadership, service planning and provisioning, financing, user and societal engagement, environmental and public health protections, and broader sanitation infrastructure that account for inequalities (Mikhael et al. 2021; Willetts et al. 2022). Further, the sanitation system is embedded in other settlement infrastructure, such as housing, roads, water and power supply. Holistic consideration of the interconnectedness of the sanitation infrastructure with the elements of the ‘sanitation system’ elements and other settlement infrastructure elements is key to progressing climate resilient sanitation (Willetts et al. 2022). Nonetheless, the design of sanitation technologies that prevent release of pathogens, despite climate hazards, remain an important aspect of a climate resilient sanitation service.

Most academic literature on the resilience of sanitation technologies focuses on centralised wastewater treatment facilities and technologies in a high income country context (Karamouz et al. 2019; Kirchhoff and Watson 2019; Neumann et al. 2015; Swanson et al. 2021). Such literature often also requires specialised modelling skills to assess resilience (Kohler et al. 2016; Olyaei et al. 2018). These modelling methods are likely technically inaccessible for many sanitation technology designers and implementers. These approaches also have limited relevance to the LMIC context where decentralised wastewater facilities are more common.

Guidance on improving the resilience of sanitation in LMIC contexts is emergent and does not include a detailed focus on technology design. Some guidance documents and academic papers detail the aforementioned systems (e.g. governance, financing etc.) needed to enable climate resilient sanitation services at a city-level (ISF-UTS and SNV, 2019; Mikhael et al. 2021; Willetts et al. 2022). Other guidance documents outline methodologies to assess climate risks to water and sanitation services and appraise adaptation options, with risks and adaptations considered in relation to overall WASH service delivery (Howard et al. 2021; UNICEF & Global Water Partnership, 2022), with sanitation technology resilience itself receiving limited analysis. Finally, guidance documents also describe risk assessment approaches to assess climate risks and provide examples of technology design adaptations (UNICEF & Global Water Partnership, 2022; USAID 2015a; World Bank 2018, 2020; World Health Organization 2022). For example, USAID (2015a) outlined a five-step process of (1) establishing context, (2) assessing vulnerability via exposure and sensitivity analysis, (3) a risk assessment based on likelihood and consequence, (4) development and comparison of responses, and (5) implementation of the response. The World Bank (2020) outlines a process of identifying threats and consequences of different climate hazards before deciding on actions, including technology changes. However, these latter resources only provide examples of modifications to sanitation infrastructure to improve resilience to climate hazards, and do not offer a systematic approach to consider the breadth of different design possibilities that could or should be considered.

There is an absence of design guidance for climate resilient decentralised sanitation technology. This heightens the risk of technology failure from climate hazards, particularly in LMICs. This research responds to this gap. The research aims to influence the integration of design features into sanitation technologies that will reduce public and environmental health risks associated with exposure to climate hazards. As part of a broader study, the research aims to develop a tool to assess the climate resilience of on-site and decentralised containment and treatment technologies in LMICs (Kohlitz et al. 2023). For example, pit latrines, septic tanks, wetland treatment systems, composting toilets, and others described in Tilley et al. (2016), as well as recent newly emerging technologies such as those under development through the Bill and Melinda Gates Foundation reinvent the toilet initiative. The research questions which form the basis of this paper are:

1. What design features enable sanitation technologies to continue to function when exposed to climate hazards?
2. What climate resilient design features from other infrastructure sectors are transferable to sanitation technologies?

Through the literature review of sanitation and infrastructure literature, we identified 25 design features that can contribute to a sanitation technology’s climate resilience. These were grouped into seven categories. The design features are relevant for sanitation technology designers and enterprises, research and development personnel, and professionals working to implement sanitation technologies in LMICs. The following sections outline the methodology used to determine the design features and categories of features, before we discuss the implications of the findings.

## Methodology

We used two literature review methods for this research, one method for sanitation literature, and another for infrastructure literature. These methods are discussed below.

### Sanitation literature

We completed a systematic review of electronic academic and grey sanitation literature. The review was consistent with PRISMA guidelines (Page et al. 2021). Information on how climate hazards affected a range of sanitation technologies was captured by the systematic review. The literature review also identified practical or theoretical features of sanitation technologies that supported its ongoing functionality despite being exposed to a climate hazard.

The ProQuest, Scopus and Web of Science core collection databases were searched for the academic literature element of the review, using combinations of the search terms shown in Table 1. All search strings were for ‘title’, ‘abstract’, and ‘keyword’ fields. Academic literature that was searched included journals, conference papers, and book chapters. Only the hazard “flood” was included in the climate string, as this hazard is ubiquitous in sanitation climate resilience literature. Other hazards or meteorological terms were not included in the search. An initial pre-screen that included intense rainfall, increase in average rainfall, drought, decrease in average rainfall, sea level rise, storm surges, cyclone/storms, wildfires, and extreme heat, returned large numbers of irrelevant records, hence the decision was made to focus the literature search. Searches purposefully included centralised sanitation technologies as well as on-site and decentralised systems. This was due to the limited literature on on-site and decentralised sanitation technology resilience, and because some centralised sanitation system resilience measures were also relevant to the research questions.

**Table 1:** Search terms for academic and grey sanitation literature

Literature type	Discipline	Search string key words
Academic	Sanitation related search string	“sanitation” OR “toilet*” OR “latrine*”, “wastewater” OR “sewage” OR “sewerage” “sanitary engineering” OR “fecal sludge” OR “faecal sludge”
Academic	Climate change related search string	“climate change” OR “flood”
Academic	Resilience related search string	“resilen*” OR “robust”
Grey literature	Sanitation and climate change	Sanitation, resilience, flood, “climate change” OR flood filetype: pdf site:.org

A grey (non-academic) sanitation literature search was limited to government and organisational reports and restricted to pdf documents, using the Google search engine. The core search string used for grey literature is also shown in Table 1. The “site” field in the table (e.g. .org in the example below) was changed to cover six domains: .org, .org.* .gov, .gov*, .edu*, .edu.*, resulting in six individual searches.

We adjusted the number of search results we reviewed based on the relevance of the results. Each google search page returned 10 results. A minimum of 20 and maximum of 50 search outputs were reviewed for each domain search. If a Google search page contained no relevant searches, no subsequent search pages were reviewed. If a page had relevant search results, we continued reviewing results until the maximum of 50 outputs were reviewed.

### Inclusion and exclusion criteria

In addition to the search filters described in Table 1 , other inclusion screening criteria for academic and grey literature included:

- a time span of January 2010 to December 2021, and English language only.
- a focus on climate-related impact on sanitation, an explicit mention of climate change was not required.
- literature about on-site sanitation technologies, sewerage, and decentralised and centralised treatment facilities.
- a focus on the physical characteristics of the sanitation technology, rather than where or how the sanitation technology is deployed or used.
- high-, middle-, and low-income country contexts.

**Table 2:** Sanitation climate resilient design categories (A – G), design features (1 − 25), and example applications

Resilience design feature and definition	Examples of applications to sanitation technologies
*A. Avoiding exposure to hazards*: Features that reduce the likelihood that critical components of the sanitation technology become directly exposed to a climate hazard.	
*1. Raising*: Raising the technology or critical components so they are less likely to come into contact with floodwater, groundwater, or rising sea levels (Azevedo de Almeida & Mostafavi, 2016; Fewster 2012; Kirchhoff and Watson 2019; Sherpa et al. 2014; Uddin et al. 2013; UNICEF & Global Water Partnership, 2022; USAID 2015a; World Bank 2020).	Pit latrine where the superstructure is raised on higher foundation or stilts to avoid floods (Islamic Development Bank 2021). Septic tank built above ground to avoid rising groundwater tables (USAID 2015a). Raising of drain/leach field for septic tanks to move above saturated groundwater (Cooper et al. 2016; World Bank 2020). Floating sanitation technologies that rise as floodwater or groundwater rises. Sensitive or hazardous equipment (e.g. electrical components such as motors, or chemicals) are raised to avoid flood waters (USAID 2017; World Bank 2020).
*2. Burying*: Installing the technology or its components underground so that they are less likely to come into contact with wind pressure, fire, or force of flooding (Schweikert and Deinert 2021).	Installing containment, treatment and disposal technologies underground (Howard et al. 2021; Luh et al. 2017; Sherpa et al. 2014; USAID 2017).
*3. Portability*: The ability of the technology to be easily moved to a new location to avoid exposure to a hazard (UNEP, 2021).	Container-based sanitation units or other forms of portable toilets that can be easily transported if needed to avoid flooding or forecasted hazards (GIZ 2021).
*4. No/low inputs*: Technologies that require little or no inputs (e.g. electricity, water, human operators) to operate, thus reducing the need for inputs that could be exposed to hazards (Abtahi et al. 2021; Howard et al. 2010; Luh et al. 2017; Schoen et al. 2015; USAID 2015a).	Sanitation technologies that continue to operate without skilled staff during or following a climate emergency. Sanitation technologies that reuse greywater (GIZ 2021; Islamic Development Bank 2021; Schoen et al. 2015; USAID 2015a, 2017). Low flush toilets and modified sewerage, including ‘condominial’ and small bore sewerage, that operate with less water than conventional sewer (GIZ 2021; Howard et al. 2010; Islamic Development Bank 2021; Luh et al. 2017; UNICEF & Global Water Partnership, 2022; USAID 2015a). Nature-based treatment technologies that continue to operate without external inputs or controls, such as constructed wetlands (Abtahi et al. 2021). Gravity-based treatment that moves waste through the system via gravity instead of pumping or other conveyance that requires energy inputs. Dry toilets, such as composting toilets and urine-diverting dry toilets, that require no electricity or water inputs, and hence can continue to function during water scarcity or power failures (Charles et al. 2010; GIZ 2021; Howard et al. 2010; Luh et al. 2017; Mills et al. 2020; Schoen et al. 2015; Sherpa et al. 2014; UNICEF & Global Water Partnership, 2022).
*B. Withstanding exposure to hazards*: Features that enable the sanitation technology to resist a climate hazard. The sanitation technology continues to function as normal (i.e. no changes in hardware or operations) even when exposed to climate hazards.	
*5. Armouring and strengthening*: Hardening or stiffening the technology or its components against a hazard (Karamouz et al. 2019; Park et al. 2013).	Concrete rings to reinforce pits in pit latrines, and sealing of ring joints, to resist pit collapse and the ingress of water into pits (GIZ 2021). Anchoring of sewer lines or tanks to the ground (Fewster 2012; Howard and Bartram 2010; World Bank 2020). Use of phase-change materials to reduce the number of freeze–thaw cycles in cold environments (Swanson et al. 2021). Submersible pumps that retain their function when inundated with water or sewage (Baca et al. 2021; World Bank 2020). Protective structures around critical components to reduce the possibility of damage by wind-borne projectiles (World Bank 2020).
*6. Oversizing*: Increasing the tolerance or capacity of the technology or its component so that it can accommodate extreme conditions, projected changes in conditions, or changes in the number of users (Park et al. 2013).	Buffer containment or buffer treatment units that can hold peak flows temporarily when large inflows occur (Olyaei et al. 2018). Large pipes that can carry higher volumes of wastewater if inflow increases during the wet season or if numbers of users increase (USAID 2015a). Temporary storage of sewage input or overflow for later treatment during times of flood, for example, via constructed wetlands (Karamouz et al. 2019). Extra storage to accommodate extra users in times of climate related migration or changes in usage patterns.
*7. Shapes that distribute pressure*: The shape of the technology creates a more uniform distribution of stress over its cross-section, thus reducing the risk of failure at weaker points (UNEP, 2021).	Construction of superstructures in geodesic shapes, instead of cubic shapes, that better distribute pressure from wind and force of flooding (UNEP, 2021). Cylindrical pits and tanks that distribute external water pressure from rising water levels. Sloped soil/drains at the foundation of the superstructure to divert runoff away from structure and prevent water from pooling, and reduce erosion risk.
*8. Circumvention*: Technology materials or designs that allow wind or water to pass through so that the stress on the technology or component is reduced (UNEP, 2021).	Waste water treatment plant with high flow bypasses to avoid damage (Batson et al. 2019; Hughes et al. 2021; Karamouz et al. 2019; Olyaei et al. 2018). Treatment facilities with porous or vegetated surfaces around the facility to absorb surface runoff, and reduce waterlogging and erosion (Azevedo de Almeida & Mostafavi, 2016; GIZ 2021; Swanson et al. 2021; USAID 2015a). Pit latrine superstructure with gaps or holes that allow wind to pass through.
*9. Sealing and barriers*: Integrating seals, barriers or other forms of protection into the technology to protect critical components or processes from being disrupted by a hazard (Curt and Tacnet 2018).	Water treatment plants with gates or barriers that prevent flood inundation or storm surge (Baca et al. 2021; Howard et al. 2010; Kirchhoff and Watson 2019, 2019; USAID 2015a; World Bank 2020). Sewer systems with shut-off valves to avoid sewer backflow during sea level rise/storm surge (Fewster 2012; Howard et al. 2010; World Bank 2020). Water-tight joins between the toilet slab and tank in UDDTs to resist flood inundation (Sherpa et al. 2014; Uddin et al. 2013). Septic tank, or decentralised water treatment systems, with non-return valves to reduce sewage backup into homes (Howard et al. 2010; Islamic Development Bank 2021; Schoen et al. 2015; Sherpa et al. 2014; UNICEF & Global Water Partnership, 2022; USAID 2015a). Sealing/waterproofing electrical equipment and pumps (Baca et al. 2021; Schoen et al. 2015; USAID 2017). Container-based sanitation that minimises exposure to floods through enclosed faeces storage (GIZ 2021; Mills et al. 2020) Insulation or shading structure to prevent excessive heat impacting treatment system or superstructure. Flame retardant insulation for critical sanitation infrastructure (Schoen et al. 2015). Sealed covers for septic tank lids (Howard and Bartram 2010; Sherpa et al. 2014).
*C. Enabling flexibility*: Features that enable the sanitation technology to be adapted or reconfigured, or have its operation changed, to continue providing sanitation services when exposed to climate hazards.	
*10. Adaptability*: The technology can be adapted or upgraded easily to function better under the changing environmental conditions (e.g. increasingly wet or increasingly dry conditions) (Howard and Bartram 2010; Mills et al. 2020).	Anaerobic digesters can be insulated to maintain a constant treatment temperature (Schoen et al. 2015). Real time monitoring of treatment inflows and quality to adjust pumps, treatment process or dosing to suit (World Bank 2020). Adjustable outlet levels to adapt treatment to changing inflows (e.g. maintain adequate water depth in constructed wetlands during dry conditions, or adjust residence time in tanks based on inflow). Pit latrines that can be readily modified based on climatic conditions (compared to a more sophisticated system), e.g. raising, or lining pits with concrete rings in instances of increased rainfall (Howard et al. 2010, 2016; Mills et al. 2020; Sherpa et al. 2014; UNICEF & Global Water Partnership, 2022). Wetland treatment systems that cope with increased effluent concentrations at times of high rainfall, and have improved operations in high temperatures (Abtahi et al. 2021).
*11. Modular design*: Additional modules can be added (plug-in type model) or removed to increase or decrease capacity of the system to accommodate variability in demand and environmental conditions (Neumann et al. 2015; Spiller et al. 2015; World Bank 2020).	Using prefabricated filtration modules (e.g. in anaerobic baffled reactors) that can be expanded as the population served by a water treatment plant grows (World Bank 2020). Including space for additional pumps in the design of an influent pump station’s concrete pad (World Bank 2020). Public toilets that share a treatment system and can have additional toilets added as demand grows. Aeration tanks in a wastewater treatment plant that can operate in parallel during times of high inflow, and allows some tanks to be disconnected during times of low inflow. Use of push-fit joints for sewage pipes rather than bolted, screwed, or glued joints, making modules easy to install and repair (Spiller et al. 2015).
*12. Platform design*: The technology shares components with other similar technologies, which makes it easier to transition between technologies to meet customer demand or prevailing environmental conditions (Neumann et al. 2015).	Sanitation technologies that use a standard toilet pan and slab (or other component) that can be transferred over from existing (obsolete or inferior) sanitation technologies. Standard pipe sizes for household wastewater conveyance. Sanitation technology that uses components (e.g. electrical equipment and pumps) that are standard for the area and follow local conventions.
*13. Redundancy and diversity*: The technology has diverse and redundant components that work in parallel, or that act as back-ups to each other. In the case of component failure(s), the back-up component enables the technology to continue functioning by performing the same functions differently (Brown 2019).	A sanitation technology that has multiple pits, tanks or treatment components that can function independently of other pits/tanks/treatment components. Toilets that can alternate between being wet or dry depending on water availability. A treatment technology that has separate processes for treating waste when inflows are very low in the dry season and very high in the wet season. Backup generators/batteries for treatment facility are available if a primary power source fails (Fewster 2012; Kirchhoff and Watson 2019; Schoen et al. 2015; World Bank 2018, 2020). Backup pumps to combat inundation from storm surge, rising sea levels, or floods (Kirchhoff and Watson 2019; Swanson et al. 2021; USAID 2015a; World Bank 2020)
*14. Signalling*: The technology, based on its standard function or by intentional design, has a way of signalling to operators or users when the sanitation technology requires modification to prevent failure or to enhance its performance (Linkov et al. 2013).	A control panel installed at a treatment facility that measures biological oxygen demand, nutrient content, etc. (Spiller et al. 2015). Flow meters to signal changes in flow rate that may require changes to operations (GIZ 2021).
*D. Containing failures*: Features that enable the sanitation technology to continue providing basic services and meet user needs despite damage to technology components caused by climate hazards.	
*15. Frangibility*: Less essential components of the technology are designed to breakaway or fail when exposed to a hazard to protect more essential components of the technology (UNEP, 2021).	Sewer manholes that burst from pressure when high flows in sewers become too great, thereby protecting the pipes.
*16. Fail-operational*: The technology can still provide its overall function even when components or processes are damaged and undergoing repair (Möller and Hansson 2008; Schoen et al. 2015).	Wastewater treatment plant that still function (at reduced capacity) if one storage pond or the drying beds are temporarily offline. Biological treatment processes that continue to treat wastewater, albeit at reduced efficiency, in response to a decrease in air temperature. An automated treatment technology with a chemical feeder that can be operated manually if electricity goes out during power shortages.
*17. Decentralisation*: Failures in decentralised systems (small-scale individual or clustered systems) are isolated locally rather than centrally (GIZ 2021; Howard et al. 2010; Sun et al. 2020; UNICEF & Global Water Partnership, 2022; USAID 2015a).	Household, on-site sanitation technologies that operate independently of neighbouring household sanitation technologies (Sun et al. 2020). Short sewer lines to decentralised treatment that reduce exposure to climate hazards compared to longer sewer lines (GIZ 2021; Luh et al. 2017; Sherpa et al. 2014). Multiple small scale sludge treatment systems that are located across a city.
*E. Limiting consequences of complete failure*: Features that minimise the negative health and environmental consequences of complete sanitation technology failure because of a climate hazard.	
*18. Safe disposal*: The materials from the destroyed technology are not toxic to the environment or public health and can be safely disposed (Linkov et al. 2013).	Materials used to build the sanitation technology can be discarded into a local landfill without posing an additional health risk to the public or the environment.
*19. Reusable materials*: The materials from the destroyed technology can be reused for other purposes, including rebuilding the technology (UNEP, 2021).	Prefabricated septic tanks (if not damaged) that can be used for other sanitation systems. Use of plastic or ferrous metal pipes that are more suitable for reuse compared to concrete pipes (Spiller et al. 2015)
*20. Fail-silence*: If the technology completely fails, it does not pose a health risk to the public or the environment beyond being unable to perform its function (Möller and Hansson 2008).	The inflow to a treatment technology can be shut off to prevent the technology from overflowing into the open environment if it has been compromised by flooding or another hazard. Pit latrines with shallower pits to reduce the risk of collapse, and limit exposure of faeces to flood waters or rising sea levels (Islamic Development Bank 2021; Sherpa et al. 2014; USAID 2015a)
*F. Facilitating fast recovery*: Features that enable the sanitation technology to be quickly rebuilt, restored, or replaced if they are damaged, disrupted or destroyed by a climate hazard.	
*21. Repair*,* restore*,* or replacement speed*: The technology and its components, processes, or operations can be quickly rebuilt, restored, or replaced if destroyed or disrupted, thereby minimising downtime or performance degradation (Hickford et al. 2018).	Junction boxes that divert the flow of waste so that damaged pipes, pits, etc. can be more quickly repaired. Biological waste treatment processes that can be quickly re-started and brought back to full capacity after being disrupted. Low-cost toilets that can be rebuilt quickly in the case of failure (Charles et al. 2010; Howard et al. 2010; Islamic Development Bank 2021; USAID 2015a). Pipe fittings that are bolted and can be isolated via shut-off valves, can be more easily replaced than a welded/glued pipe without isolation. Filter media in treatment components that can be easily swapped out if it becomes clogged. Water treatment plants where electrical equipment is built to local standards with locally available materials (e.g. wiring and voltages that follow local convention). Shallow burial depths for sewers to help find and repair leaks (Schoen et al. 2015). “Flat-pack” type sanitation technologies that can be transported and constructed quickly in areas impacted by climate hazards. Sanitation technologies housed in shipping containers that can be transported and deployed in areas where sanitation technology has been damaged (Shyu et al. 2021; University of South Florida 2021)
*22. Accessibility for rapid flaw detection and repair*: Components or processes of the technology can be easily accessed for examination and repairs (Mottahedi et al. 2021).	Inspection chambers that allow operators or repairers to see inside containments. Manholes/hatches that can be easily opened for inspectors/repairers to conduct examinations or make repairs. Above ground tanks that can be easily repaired in case of leakage (compared to an underground tank). Shallow burial depth of drainage channels or pipes that simplify finding and repairing of leaks, hence reducing the repair cost and time (Schoen et al. 2015).
*G. Supporting resilience beyond sanitation services*: Features that enable the sanitation technology to provide other benefits to people or systems that aid in broader community or system resilience.	
*23. Reciprocity*: Through operations, the technology also builds resilience in, or aids, another on-site or off-site system (Brown 2019).	Treated sludge or wastewater that can be used in agricultural applications (USAID 2015b). Biogas from treated waste can be used for electricity production and use off-site. Treated waste that is made into briquettes for fuel.
*24. Hybridising*: Unrelated systems or technologies share the same physical space or structure, thus saving space and enhancing opportunities for reciprocity (Brown 2019).	A biogas system that provides a source of on-site energy for the treatment system.
*25. Transformative capacity*: The technology provides an additional service(s) beyond its usual intent that further aids resilient communities (Brown 2019).	A sanitation technology that can also accept food or other solid waste (e.g. co-composting).

Exclusion criteria included:

- literature that focused on sanitation services related to other parts of the sanitation chain such as emptying, or wider services including drainage, or solid waste management such as rubbish management, and resilience to non-climate related events (e.g. COVID-19; terrorism; civil unrest).
- literature concerned only with the mitigation of greenhouse gas emissions.
- literature that was deemed irrelevant for other reasons. For example, documents that focused on sanitation supporting systems or the sanitation enabling environment without reference to technological design.

### Processing and screening

Literature results were imported into the Zotero reference management software and then screened based on the criteria above. The number of documents included and excluded at each screening stage was recorded in the PRISMA template shown in Supplementary Material, Figure S1, Section S1.

Screening was completed by two of the authors of this article (IC and JK), who each have over fifteen years’ experience in the water, sanitation, and hygiene sector. The first round of screening removed duplicates. A second screening round used the criteria listed above to review each documents’ title and abstract, title and summary, or title and conclusion. The researchers used the Abstrackr software platform to complete the screening independently. Literature was assessed in Abstrackr as ‘relevant’, ‘not relevant’ or ‘maybe relevant’. After completion of the first screening round, the research team addressed screening conflicts or ‘maybe relevant’ labels through consultation, and, where needed, via a full text review.

Full document texts were reviewed in a third round of screening. IC reviewed full-texts and screened documents using the inclusion and exclusion criteria outlined above. Additional documents were identified via hand search, and after screening, relevant texts were included in the review. These included seven additional academic papers (including two published soon after the search was completed) and two grey literature documents. The various screening processes led to the inclusion of 40 academic papers and 16 grey literature documents.

As part of the review process, a list of climate events and climate trends and associated specific hazards for sanitation technology was compiled, these are included in Table S1 of the Supplementary Material.

### Non-sanitation literature

A review of literature on the resilience of technologies and infrastructure in other sectors (‘non sanitation literature’) was also conducted. It aimed to identify design concepts or principles that enhanced resilience and could be transferred to sanitation technologies. This review was exploratory, it continued until no new concepts or principles were identified over multiple searches. Hence, it aimed to extract key concepts or principles that are repeated in literature on technological resilience, rather than comprehensively reviewing this literature.

Based on consultations with peers experienced with climate resilient technology, and rapid online searches of resilient technologies, we sought relevant academic and grey literature from five technology-dependent sectors: water supply, waste-to-energy, software/computing, energy, and housing. The review also included general theoretical literature on the resilience of technologies.

Searches were completed using ProQuest academic database and Google. ProQuest search strings and their results are shown in the Supplementary Material, Section S2, Table S2. The most productive search strings were entered in Google Scholar and normal Google searches. Forward and backward citation searching of selected literature was also done to explore the literature further. Documents that appeared to introduce a concept or principle for resilient technologies that had not been captured by previous documents in the non-sanitation literature review, or had a different perspective on a concept or principle already captured, were saved for further review. In total, we saved 56 academic and grey non-sanitation documents.

### Analysis and coding: design features

Once screening was complete, sanitation and non-sanitation literature was coded using NVivo software. Sanitation related literature was coded for context (urban/rural), country focus (low, middle, and, or high income country focus), the presence or absence of a framework to assess resilience, and where relevant, the method used in the literature to assess resilience (i.e. qualitative, quantitative, mixed methods, or not applicable).

An inductive coding approach was used to extract text fragments and code sanitation and non-sanitation text. Coding was based on the research questions discussed in the introduction. Codes were assigned to text which identified design features that either positively or negatively affected the climate resilience of sanitation technology, text that described a concept or principle claimed to enhance the resilience of technology or infrastructure, and real-world examples of implemented sanitation technology that accommodated climate impacts.

As discussed earlier, the scope of this research focused on the technology design only. Other aspects related to the technology, for example where and how the sanitation technology is deployed, though important, were not part of the scope. In addition, aspects that impact technology selection but go beyond the technology design itself were excluded. For example, affordability, acceptability, capacity to maintain the technology, and accessibility, though critical to the sustainability of a sanitation technology in a LMIC, were also not included in the scope.

Codes were then used to identify themes of resilient design features. For example, Uddin et al. (2013) described how the faeces vault in urine diverting dry toilets (UDDT) protect the sanitation technology from floods. Hence, relevant text fragments in this article were coded under a theme of ‘sealing’. These themes were developed by IC and JK independently. JK reviewed the non-sanitation literature codes, and IC reviewed the sanitation literature. Once the themes were developed, the researchers convened to create a preliminary design feature list. A further inductive round of coding of texts was completed when needed. Design features were either added or consolidated in this final round. A definition for each feature was then added by the authors, and examples of the feature in practice was added to illustrate the feature. In the table below, examples that are based on the authors’ experience do not have a citation. The feature examples were sourced from the reviewed literature, or from the authors’ experience.

Once features had been outlined, we grouped features with those that had similar conceptual approaches to how they improved resilience in response to hazards. This resulted in seven author-defined conceptual categories, these are shown in the results section as headings ‘A’ to ‘G’ (e.g. avoiding exposure to hazards, withstanding exposure to hazards, etc.). Each of these is based in theories relevant to resilience, including disaster risk theory, socio-ecological systems theory and safety engineering theory. For a more detailed account of the relevance of these theories in the sanitation and water sector see Kohlitz et al. (2017).

## Results

From the literature review and analysis, we identified and defined 25 key design features that contribute to sanitation resilience and provided practical examples of each feature. These design features are grouped into seven categories based on climate adaptation theory and literature. Below we describe the basis for the categories and then present the features themselves with illustration of their application to sanitation technologies.

The seven categories relate to different aspects of climate resilience, each employing different strategies to reduce the likelihood, magnitude, length or consequences of service disruptions or build on potential opportunities for co-benefits. The first category of ‘avoiding exposure to hazards’ includes design features that reduce the likelihood of sanitation technology critical components of the becoming exposed to a climate hazard. This category is aligned to climate and disaster risk theory that proposes that vulnerability of or risk to a system can be reduced through reducing the exposure of the system to hazards (Gallopín 2006; Smit and Wandel 2006). The second category is ‘withstanding exposure to hazards’. This includes features that enable the sanitation technology to resist a climate hazard and continue to function as normal (i.e. no changes in hardware or operations) even when exposed to climate hazards. According to climate and disaster risk theory, reducing system vulnerability involves decreasing its sensitivity to hazards (Bruneau et al. 2003; Smit and Wandel 2006). The third category, ‘enabling flexibility’, refers to features that allow the sanitation technology to be adapted or reconfigured, or have its operation changed, ensuring continued service during exposure to climate hazards. Flexibility is consistent with social-ecological systems theory which proposes that system resilience can be enhanced through improving the ability of the system to reorganize itself when exposed to disturbances (Walker and Salt 2012).

The fourth category is ‘containing failures’. Features within this category ensure that sanitation technologies can continue to provide basic services and meet user needs, even if components are damaged by climate hazards. This category aligns with social-ecological systems theory ideas. Namely, that system resilience can be enhanced through managing the characteristic of ‘connectivity’ – the manner through which materials interact within a system – so negative effects are isolated and not propagated across the whole system (Biggs et al. 2012). The fifth category, ‘limiting consequences of complete failure,’ focuses on features that mitigate negative health and environmental impacts when sanitation systems fail entirely due to climate hazards. Safety engineering theory proposes that system failure is often inevitable and systems should be designed to fail safely (Möller and Hansson 2008). The sixth category, is ‘facilitating fast recovery’. It includes features that enable the sanitation technology to be quickly rebuilt, restored, or replaced if they are damaged, disrupted or destroyed by a climate hazard. This category is aligned to disaster risk theory, which notes that system resilience can be enhanced through reduced time to recovery (Bruneau et al. 2003). Finally, the seventh category ‘supporting resilience beyond sanitation services’ refers to sanitation technology features that offer additional benefits to people or systems that aid in broader community or system resilience. According to social-ecological systems theory, the resilience of any system depends on the resilience of interconnected systems it interacts with (Folke et al. 2010).

We present the 25 design features (numbered 1–25) in Table 2, each aligned to one of the seven categories (labelled as A-G). In addition, examples of the application of each design feature for different sanitation technologies illustrates their application in practice.

## Discussion

In the following discussion, we situate the design features and their relevance in the existing sanitation resilience literature. We also critically reflect the design features utility and limitations.

### Situating the design features in technological resilience literature

Global literature on technological resilience tends to conceptualise resilience in terms of the oft cited ‘4 Rs’ of engineering resilience. These are: (i) robustness (the ability to withstand shocks without loss of function), (ii) rapidity (the time required to return to functionality), (iii) resourcefulness (the ability to mobilise resources), and (iv) redundancy (the ability to substitute components) (Bruneau et al. 2003; Chambers et al. 2022). Our 25 features shown in Table 2 do not reflect the ‘resourcefulness’ concept of the ‘4 Rs’ because this is a characteristic of the management entity and processes, not the sanitation technology, because of this, it was beyond the scope of this study although we acknowledge ‘resourcefulness’ importance for resilience. Though the design features in Table 2 are consistent with concepts of robustness, rapidity, and redundancy, these design features build on and expand on what constitutes sanitation technology resilience to climate hazards.

Robustness is the most cited category in the literature. We further distinguished robustness to two categories shown in Table 2, ‘avoiding exposure to hazards’ and ‘withstanding exposure to hazards’. This distinction is important because of the implications for design considerations. Design features associated with ‘withstanding exposure to hazards’ are concerned with construction approach and material types that are used in the sanitation technology. Meanwhile, features associated with ‘avoiding exposure to hazards’ are dependent on the technology’s operation and the context where the technology will be deployed.

These two categories and associated resilience design features were the most cited in the sanitation literature we reviewed. Similarly, Chambers et al. (2022) found robustness to be the most frequently cited resilience feature for sanitation systems. This is perhaps a result of the sanitation sector’s proclivity to focus on damage caused by climate hazards to sanitation structures as opposed to the disruptions to sanitation operations and processes (Hyde-Smith et al. 2022). That is, it is easier to imagine solutions that involve resisting changes in environmental conditions, compared to technology solutions that accept and accommodate or adapt to those changes and associated disruptions.

Like Chambers et al. (2022), we found literature stressed the importance of the two R’s of ‘redundancy’ and ‘rapidity’, as well as ‘adaptability’ as important resilient design features. However, as seen in Table 2, we took a different categorisation approach to these features when compared to existing literature. For instance, under the category ‘enabling flexibility’ we grouped features of ‘redundancy’ and ‘adaptability’, along with ‘modular design’, ‘platform design’, and ‘signalling’. The category of ‘enabling flexibility’ expands beyond the ‘4 Rs’ concepts. It acknowledges more recent advancements in resilience thinking that consider accommodating rather than simply resisting environmental changes, an important approach to climate change resilience. Meanwhile, we conceptualise ‘rapidity’ in Table 2 as inclusive of our ‘repair, restore or replacement speed’ and ‘accessibility for rapid flaw detection and repair’ features. These features were included in the ‘facilitating fast recovery’ category, to distinguish between design features where a component can be easily fixed or restored, and features that enable a component to be easily reached for quick maintenance.

Beyond the 4 R’s, we propose three additional categories of resilient design features of ‘containing failures’, ‘limiting consequences of complete failure’, and ‘supporting resilience beyond sanitation services’. These categories were identified in the literature review, but are not captured in the ‘4 Rs’ or in Chambers et al.‘s (2022) review of sanitation resilience literature. We found relatively few examples of design features from these additional categories in the sanitation literature. Although these categories were primarily derived from other technology-dependent sectors, we posit they are also relevant to resilient sanitation technology design.

The ‘containing failures’ and ‘limiting consequences of complete failure’ categories acknowledge that it is unrealistic to expect that sanitation technologies can be designed to never fail, particularly in the context of climate change. Consequently, the management of failures is an important aspect of resilience. Finally, our inclusion of ‘supporting resilience beyond sanitation services’ reflects arguments in the literature that the resilience of specific technologies and systems is often intertwined with the resilience of linked systems (Brown 2019). Sanitation can provide additional benefits that support societal resilience, and these benefits can also act as additional drivers to support investment in sanitation services.

Beyond the 4 R’s, Sherpa et al’s (2014) review paper also analysed the vulnerability and adaptability of generic sanitation technology types. The paper analysed technology categories such as waterless systems, and pit-based systems. However, this categorisation does not support design thinking beyond the pre-determined, conventional technology types listed in Sherpa et al’s paper. It also does not draw on resilience concepts from other non-sanitation technology-dependent sectors. As discussed earlier, we found non-sanitation technology literature an important source of resilience concepts that are relevant to sanitation applications.

As noted in the introduction, risk-based approaches to resilience also featured in the reviewed literature. These are approaches that assess exposure to climate hazards, assess risk to sanitation technology based on likelihood and consequence, and outline responses (USAID 2015a; World Bank 2018). We view the design features in Table 2 as additive to these approaches to resilient sanitation design. As is discussed below in the next sub-section, the design features can be used to inform the assessment of a current technology’s exposure to climate risk and inform new technology or modifications to existing sanitation technology.

### Application and adaptation of the design features

The features can aid the design of climate resilient on-site and decentralised sanitation technology. Based on the 25 design features, a practitioner-based tool called ClimateFIRST (Climate Framework to Improve the Resilience of Sanitation Technologies) has been developed to assess the climate resilience of different sanitation technologies (Kohlitz et al. 2023). It incorporates the features within a wider process of consideration of a range of key climate hazards and expected impact of these on existing or technologies currently in design. ClimateFIRST prompts the user to consider the type and components of the technology which are in and out of scope to assess. As the specific local design and application of technology types can differ, the tool recommends the features are best applied to specific sanitation technologies and not a generic technology (e.g. a specific septic tank construction design rather than septic tanks in general).

The features also have standalone merit. Existing sanitation technologies can be evaluated or compared based on the presence or absence of design features. The resilient design features and examples may stimulate critical thinking of sanitation technology design, improved designs of sanitation technology, and more critical selection of a sanitation technology for a specific context. For example, ‘no/low inputs’ may prompt consideration of low-flush toilet designs, to adapt to times of drought. Or, ‘repair speed’ may encourage the use of locally available materials in sanitation technology selection or design to foster rapid recovery after climate change induced failures.

The design features could have potential to contribute to future standards in sanitation technology design. Product standards are instrumental for ensuring that novel sanitation technologies have good quality and performance, but careful thought is needed to consider the interplay between standardisation and other sustainability aspects (Cid et al. 2022). Further research could shed light on the feasibility of addressing climate hazards in sanitation standards, such as the International Organization for Standardization (ISO) standards for non-sewered sanitation (2018), through consideration of which features outlined in this paper are amenable to standardisation.

The design features may have applicability to technologies beyond sanitation. As discussed in the methodology, the literature review drew from other disciplines such as building design, in developing the list of design features. The design features are generalised, and hence likely applicable to other infrastructure besides decentralised sanitation technologies in LMICs. However, any relevance of the 25 design features would need to be examined in more detail.

### Critical considerations when applying the resilient design features

There are several critical considerations in the choice of which design features to implement and to what extent to implement the features. This includes which climate hazards are being designed for since the features are not universally applicable, weighing of benefits and costs, consideration of trade-offs between different features and the impact of uncertainty in climate change and related risk tolerance. These complexities then demand an inclusive approach to weighing up appropriate design features, and situating such choices within the wider service delivery context. Below we expand on each of these points.

The features are not universally applicable to all sanitation technology and every LMIC context. For example, ‘burying’ to avoid wind forces is irrelevant to a container-based sanitation pedestal that sits inside a house. The relevance of features is also dependent on the social, economic, and geographical context a sanitation technology is being implemented in. Such as, raising a sanitation technology (or its outlets) to avoid floodwaters is only applicable in locations where flooding is a risk. Similarly, signalling technology is relevant only where there are people, such as users or operators, that have the capacity to view and respond to the signal. As such, it may not always be applicable in low-resource settings in LMICs.

Sanitation technology designers and implementers need to critique the costs and benefits of adding new design features to a sanitation technology. The absence of certain design features does not necessarily indicate a climate vulnerable sanitation technology. In addition, including too many features in a sanitation technology is likely to result in an overdesigned, expensive, and impractical sanitation technology. Some features may be more expensive or impractical to integrate into a sanitation technology, when compared to other features or the status quo. For example, implementing real time electronic flow rate monitoring to a decentralised, anaerobic batch reactor systems, though beneficial, may be cost prohibitive and not relevant to a LMIC context.

Resilience trade-offs need to also be considered with the integration of any of the design features. Adding a design feature may improve resilience against one hazard but compromise resilience against another hazard. For example, raising a sanitation technology against floods then could expose it to wind forces. Using cheaper materials that are faster to rebuild may compromise their robustness against hazards. While “oversizing” wastewater inflow pipes for a wastewater treatment facility may increase resilience as higher volumes of wastewater can be carried during the wet season. However, in times of drought, low flows may result in sedimentation and blockages in oversized pipe.

Adding more features may also present a trade-off with the appropriateness or relevance of a sanitation technology. For example, raising a latrine may reduce flood impacts. However, it also impacts the latrine’s accessibility for users living with a disability. Further, new sanitation technology designs may be not accepted by users, or new features may increase the level of management or maintenance needed for the sanitation technology to a point which makes it impossible to maintain. These examples point to the need for a wider consideration of costs and benefits of features, beyond simply an analysis of climate resilience.

As discussed earlier, sanitation technology is interconnected with a broader system that can impact the resilience of a sanitation technology. For example, raising a public toilet block may reduce the impact of flood hazards on the toilet. In the event of a flood, the toilet’s access path could be washed away resulting in the toilet being inaccessible and unused. Those using the design features should be clear on the boundary of their resilience assessment, and the implications of climate hazards beyond this boundary.

Decision-making by sanitation designers and implementers is further complicated by the uncertainty of climate change, a key aspect of climate change adaptation and resilience that is often overlooked (Kohlitz et al. 2019). It is difficult to predict the frequency and severity of future climate hazards at local scales. Climate uncertainty means the benefit of new features is uncertain. Sanitation actors are faced with the difficult decision of investing in more costly, resilient designs that may not be needed. Equally, if design features are not incorporated unacceptable levels of public and environmental harm may eventuate. For this reason, decision-makers will need to consider the tolerance of sanitation failure risk they are comfortable with in improving sanitation design. Lastly, although uncertainty can be accommodated to some degree through flexible technology design (e.g. the ‘enabling flexibility’ features), in addition, flexible management and governance institutions are critical (Chester and Allenby 2021). Hence, handling uncertainty is about more than technology design.

Given these many aspects, an inclusive process involving diverse people in making design choices is likely to enable better choices, rather than sanitation technology designers considering these dimensions in isolation from application contexts. In particular, including gendered perspectives is important as these considerations are not purely technical (Kumar et al. 2023). Further, the design and implementation of sanitation technology should be part of a broader process that considers planning, institutions, financing, management options and approaches *(i.e. ‘resourcefulness’ of the 4 Rs)*, user awareness, water cycle management and monitoring and evaluation. Such processes help ensure that technology adaptations are supported by a broader enabling environment (Willetts et al. 2022).

## Conclusion

Sanitation technologies, as part of wider sanitation service delivery systems, are at risk from climate change events and trends. Failing sanitation services compromise public health and cause environmental pollution. The risks to sanitation technology are heightened by the lack of guidance for sanitation designers and implementers on how best to integrate climate change considerations into their designs.

This study aimed to identify design features that enhanced the climate resilience of sanitation technology. Through a review of academic and grey sanitation and infrastructure literature, we identified 25 such design features. These features include seven groups of varying strategies, including: avoiding exposure to hazards, withstanding exposure to hazards, enabling flexibility, containing failures, limiting consequences of complete failure, facilitating fast recovery, and providing benefits beyond sanitation technology resilience. The design features build on and expand on what constitutes sanitation technology resilience to climate hazards.

The design features can help sanitation actors reflect on the potential impacts of climate change on sanitation technology, and make informed decisions about the design and implementation of the technology in different contexts. For instance, the design feature list can aid in comparison of different sanitation technologies based on the presence or absence of specific design features. The features could also prompt changes in the design of new or existing sanitation technology to improve their resilience to climate change. They may also contribute to future iterations of standards related to sanitation technology in LMICs.

As the features are novel, they are yet to be comprehensively tested by diverse sanitation actors. The research team and sanitation designers have completed a limited set of assessments of sanitation technologies, including both typical technologies found in LMICs as well as advance technologies intended for application in LMICs in the future. An example assessment of an anaerobic baffled reactor in Indonesia is included in the Supplementary Material, Section S4 (Kohlitz et al. 2023; UTS-ISF 2023). It is expected that further applications, including across diverse contexts could iteratively evolve the 25 features described in this paper. The ease of understanding and relevance of design features could also be refined through further testing and feedback by sanitation designers and implementers in LMICs. In particular, from a practical perspective, this could include refining the features to a subset that are most feasible, affordable, and simplest to implement in LMIC contexts. Further testing is also needed to assess the effectiveness of the design features to reduce service disruptions or limit health consequences of failure. In addition, the features could be expanded based on a consideration of a greater range of hazards. Flood related hazards dominated the current sanitation resilience literature at the time of writing this paper, which may have led to omission of important design features relevant toother climate hazards. Finally, we agree with other research that argues supportive institutional, financial, economic, and social structures need to be considered along with technology considerations. The design features identified in this article are techno-centric, and could benefit through integration with other frameworks and decision-making processes that consider a more holistic approach to sanitation.

## Supplementary Information

Below is the link to the electronic supplementary material.

ESM 1 (DOCX 82.0 KB)

## Data Availability

Not applicable.
