# Supplementary material for: Designing for climate change: twenty-five design features to improve sanitation technology resilience in low- and middle- income countries
Source: Mitig Adapt Strateg Glob Chang. 2024 Nov 13;29(8):82. doi: 10.1007/s11027-024-10177-7 (PMC11561023; doi:10.1007/s11027-024-10177-7)
Supplement: Supplementary file 1 — (DOCX 82.0 KB) [file 11027_2024_10177_MOESM1_ESM.docx]

# Supplementary material

**Article title**

Designing for climate change: Twenty-five design features to improve sanitation technology resilience in low- and middle- income countries

**Journal**

Mitigation and Adaptation Strategies for Global Change

**Authors**

Ian Cunningham, Jeremy Kohlitz, Juliet Willetts

# Sanitation literature search PRISMA diagram

**Identification of studies via other methods**

**Identification of academic studies via databases**

Records removed *before screening*:

Database duplicate records removed (n =670)

Database records removed for other reasons (n = 263)

Google duplicate records removed (n = 5)

Google records removed for other reasons (n = 131)

Records identified from:

Hand search (n = 9)

Records identified from*:

Databases (n = 1795)

Google/grey lit (n = 177)

**Identification**

Records screened

Databases (n = 405)

Google/grey (n = 41)

Records excluded**

Databases (n = 351)

Google/grey (n = 26)

Reports sought for retrieval

Databases (n = 44)

Google/grey (n = 14)

Reports not retrieved

(n = 0)

Reports sought for retrieval

(n = 9)

**Screening**

Reports not retrieved

(n = 0)

Reports excluded:

Reason 1.1 (n = 5, same study repeated in papers/conferences)

Reason 1.2 (n = 4, poor quality paper/conference)
Reason 1.3 (n = 2, paper/conference focuses on water supply)

Reason 2.1 (n = 1 same study repeated in multiple reports)

Reports assessed for eligibility

Databases (n = 33)

Google/grey (n = 14)

Reports assessed for eligibility

(n = 9)

Reports excluded (n = 0)

Studies included in review (n = 47)

Reports of included studies (n = 9)

**Included**

**Figure S1:** PRISMA 2020 flow diagram for new systematic reviews which included searches of databases, registers, and other sources
From: Source Page et al. (2021)

# Climate events and associated hazards

Table S1 below shows the range of climate events and the relevant hazard events to sanitation technology that were considered for the study. The hazard events were based on literature reviews that described climate event impacts on sanitation technology, and those inferred by the authors based on their experience in the water and sanitation sector.

**Table S1:** Climate events and associated hazards for sanitation technologies

| Hazardous events and trends (HET) | | | | | | | | |
| --- | --- | --- | --- | --- | --- | --- | --- | --- |
|  | **Floods**  Fluvial flooding (overflowing of a river or other water body) and pluvial flooding (precipitation intensity exceeds drainage capacity) | **Changing precipitation patterns**  Increased variability in seasonal precipitation patterns and inter- annual precipitation | **High sea level**  Permanent inundation from sea level rise or temporary seawater intrusion/coastal flooding due to sea level rise, storm surge, high tide or wave setup | **Fire weather**  Weather conditions (temperature, soil moisture, humidity, and wind) that trigger and sustain fires | **Severe wind**  High wind velocity due to thunderstorms, wind gusts, tornadoes, or cyclones | **Drought**  Episodic combination of low rainfall and runoff deficit, and evaporation that leads to dry soil (i.e. hydrological drought) | **Changing air temperature**  Increased variability in diurnal and seasonal air temperature | **Extreme heat**  Episodic high surface air temperature events that are potentially exacerbated by humidity |
| **Hazards** | Landslides Erosion  Force of flood waters Increased inflow velocity Increased inflow volume  Increased levels of receiving waterways  Rise in groundwater level and/or groundwater saturation  Water ingress/inundation  Changes in pathogen concentration in inflow  Disrupted access to sanitation technology for O&M  Disrupted access to sanitation technology for major repairs  Disrupted electricity inputs to sanitation technology  Disrupted faecal sludge emptying services  Disrupted water inputs to sanitation technology | Expansion/ contraction of soils  Rise in groundwater level and/or groundwater saturation  Variable inflow velocity  Variable inflow volume | Corrosion Erosion  Expansion/contraction of soils  Force of flood waters Increased inflow velocity Increased inflow volume  Rise in groundwater level and/or groundwater saturation  Water ingress/inundation  Biological organisms exposed to saltwater  Disrupted access to sanitation technology for O&M  Disrupted access to sanitation technology for major repairs  Disrupted electricity inputs to sanitation technology  Disrupted faecal sludge emptying services  Disrupted water inputs to sanitation technology | Exposure to flames  Temperature driven expansion/ contraction of materials  Disrupted access to sanitation technology for O&M  Disrupted access to sanitation technology for major repairs  Disrupted electricity inputs to sanitation technology  Disrupted faecal sludge emptying services  Disrupted water inputs to sanitation technology | Uprooting by fallen trees  Wind force on sanitation structures  Wind-blown debris  Disrupted access to sanitation technology for O&M  Disrupted access to sanitation technology for major repairs  Disrupted electricity inputs to sanitation technology  Disrupted faecal sludge emptying services  Disrupted water inputs to sanitation technology | Corrosion  Expansion/ contraction of soils  Reduced inflow velocity  Reduced inflow volume  Changes in pathogen concentration in inflow  Reduced dilution capacity of receiving waters  Disrupted water inputs to sanitation technology | Expansion/ contraction of soils  Temperature driven expansion/ contraction of materials  Extreme heat  Variation in inflow or storage temperature | Temperature driven expansion/ contraction of materials  Extreme heat  Variation in inflow or storage temperature  Disrupted electricity inputs to sanitation technology |

Source: (Kohlitz et al., 2023)

# Literature review on technological resilience to climate change outside of sanitation

**Objective:** Draw out key lessons/concepts/principles on designing distribute technologies to be resilient to climate change from non-sanitation fields that may be transferrable to sanitation technologies.

**Analogue technologies:** Literature on the following groups of technologies will be explored to understand if they have lessons/concepts/principles for climate resilient design that are transferrable to sanitation technologies (or contain generic theory about resilience of technologies in general to shocks and disturbances in general):

- Small-scale water supply;
- Decentralised waste-to-energy;
- Software/computing;
- Decentralised energy technologies; and
- Housing

**Literature searching:** Table S2 below list the results of searches in ProQuest into the analogue technology spaces, as well as general searches. In addition to exploring the results of these search strings, the most productive search strings were entered into Google Scholar and normal Google searches.

Forward and backward citation searching was done on relevant papers.

**Table S2:** ProQuest probing search results for analogue technology

| **Water supply** | | |
| --- | --- | --- |
| **Materials** | **Search terms** | **# of results** |
| All | ab("climate change") AND ab("resilien*") AND ab("technolog*") AND ab("water supply") | 37 |
| Peer-reviewed only | ab("climate change") AND ab("resilien*") AND ab("technolog*") AND ab("water supply") | 19 |
| Peer-reviewed only | ab("climate change") AND ab("resilien*") AND ab("technolog*") AND ab("water") | 250 |
| All | ab("climate change") AND ab("resilien*") AND ti("technolog*") AND ab("water") | 43 |
| All | ab("climate change") AND ab("resilien*" OR “robust” OR “resist*” OR “climate-proof*” OR “flexible” OR “adaptive” OR “responsive” OR “sturdy”) AND ti("technolog*") AND ab("water") | 100 |
| Peer-reviewed only | ab("climate change") AND ti("technolog*") AND ab("water") | 372 |
| **Waste to energy** | | |
| Peer-reviewed only | ab("climate change") AND ab("technolog*") AND ab("waste to energy") | 36 |
| All | ab("climate change") AND ab("waste to energy") | 233 |
| All | ab("climate change") AND ab("energy from waste") | 81 |
| **Software/computing** | | |
| All | ti("software") AND ti("resilience") | 197 |
| Peer-reviewed only | ti("software") AND ti("resilience") | 21 |
| **Energy** | | |
| Peer-reviewed only | ab("energy") AND ab("resilien*") AND ti("technolog*") AND ab("climate change") | 27 |
| Peer-reviewed only | ab("energy") AND ab("resilien*") AND ab("technolog*") AND ab("climate change") | 205 |
| Peer-reviewed only | ab("energy") AND ab("resilien*" OR “robust” OR “resist*” OR “climate-proof*” OR “flexible” OR “adaptive” OR “responsive” OR “sturdy”) AND ti("technolog*") AND ab("climate change") | 87 |
| **Housing** | | |
| Peer-reviewed only | ab("hous*") AND ab("resilien*") AND ab("climate change") | 588 |
| Peer-reviewed only | ab("hous*") AND ab("resilien*") AND ab("climate change") NOT ab("household") | 174 |
| Peer-reviewed only | ti("hous*" OR "home*") AND ab("resilien*") AND ab("climate change") NOT ab("household") | 48 |
| Peer-reviewed only | ti("hous*" OR "home*") AND ab("resilien*" OR “robust” OR “resist*” OR “climate-proof*” OR “flexible” OR “adaptive” OR “responsive” OR “sturdy”) AND ab("climate change") NOT ab("household") | 118 |
| **General search terms** | | |
| Peer-reviewed only | ab(“resilien* engineering") | 247 |
| All | ab("engineering resilien*") | 166 |
| All | ab("technolog* resilience") | 32 |
| Peer-reviewed only | ab("resilien*") AND ti("technolog*") AND ab("framework" OR "criteria" OR "assess*") | 421 |
| Peer-reviewed only | ab("resilien*") AND ti("technolog*") AND ab("framework" OR "criteria") | 95 |

# Example assessment of aerated batch reactor in Banjarmasin, Indonesia

Table S3 below shows the output of an assessment of an anaerobic baffled reactor (ABR) in Banjarmasin, Indonesia. The full assessment, including the relevant hazards, their possible impacts, existing climate resilient features of the ABR, and an overall resilience assessment can be found in the full ABR assessment on via the ClimateFIRST website (UTS-ISF, 2023).

**Table S3:** Climate-related risks and design feature improvements for an ABR

| **Design Feature and definition** | **Climate Related Risks** | **Improvements** |
| --- | --- | --- |
| **Armouring and Strengthening**  *Hardening or stiffening the technology or its components against an expected force.* | It's possible it could crack under soil expansion and contraction, or through heavy traffic on the surface of the ABR. It's unknown what level of soil expansion/contraction would do this, however, ensuring it's installed in unreactive soils would help. These systems are may be located at the edge of a waterway (i.e. river bank) or natural low point in an area (i.e. on coast). It is therefore prone to impact of floodwaters (possibly storm surge in other locations if located at the beach) and may require extra armouring in these contexts. | The strength of the armouring and strengthening is also dependent on the quality of the concrete (e.g. rebar, water quality, concrete mix) and installation. As it's not pre-fabricated it's dependent on the skills/diligence of the installer. Pre-fabricated concrete modules could achieve armouring and reduce this risk, however, it comes with transport challenges (and also means local masons are not involved in construction). As per 'weak point' comment, extra armoring may be relevant if located adjacent to a waterway or coastal area. |
| **Oversizing**  *Increasing the tolerance or capacity of the technology or its component so that it can accommodate extreme conditions, projected changes in conditions, or changes in number of users.* | The ABR is designed for 20L/capita/day and has a peak design factor of 4. Once installed in a place it is difficult to expand. It has some capacity to cope with 'spikes' in usage. However, prolonged usage or a permanent increase in the number of users will mean it reaches its capacity. | Increasing the buffer capacity of the first chamber may be an option if there is adequate space. Allowing space for additional buffer chambers to be added to the end may be a consideration in the siting of the ABR |
| **Shapes that distribute**  *The shape of the technology creates more uniform distribution of stress over its cross-section, thus reducing the risk of failure at weaker points.* | The square shape could be problematic with tidal/wave/flood forces on edges. | For ABRs that are located on the edge of a waterway the shape could ensure there are no edges/protrusions in the waterway that could create increased pressuring during high river flows. |
| **Sealing and barriers**  *Integrating seals, barriers or other forms of protection into the technology to protect critical components or processes from being disrupted by a hazard.* | The outlet is not sealed, so backflow is possible when water levels are high | You could install a non-return valve on the outlet to prevent backflow from entering chambers Screens for input could reduce clogging from solid waste or other debris. This may also introduce another failure point and also introduces another level of maintenance moving potential downstream failure (clogging over time) upstream (more shorter term challenges such as blocked screens). |
| **Adaptability**  *The technology design can be adapted or upgraded easily to function better under the changing environmental conditions (e.g. increasingly wet or increasingly dry conditions).* | The technology needs a minimum amount of water to ensure it can flow between chambers. Reductions in water supply (e.g. drought) would likely reduce water flow potentially reducing its functionality. Adaptability in this regard is not integrated into the design. It would require some user intervention.  In times of increasing flood or rainfall, there may be additional water inputs into the ABR. If retention times were too fast, this would compromise the level of treatment.  Discharge to a soak pit (not detailed in the design) could reduce the impact of backflow from raised receiving water levels and may reduce impact on waterway quality during drought periods when dilution capacity of waterways are reduced. | Effluent could be recirculated through the ABR in times of low water. This would require a pumping system which adds cost and complexity to the design.  A shut off valve or similar may be useful in times of flood, sea inundation or high rain if water ingress into the ABR is an issue. |
| **Modular design**  *Additional modules can be added (plug-in type model) or removed to increase or decrease capacity of the system to accommodate variability in demand and environmental conditions.* | Module elements (e.g. additional chambers) to the ABR can not be easily removed or bypassed, however could be added if there was space at either end or possibly piped to additional baffles nearby. However, adding on extra modules is not a common practice. | The concrete structure makes removing modules difficult. Providing pipe fitting or junction boxes at the start or end would ease future modifications, as would allowing additional space for more buffers or a parallel systems in the site selection |
| **Signalling**  *The technology, by the nature of how it functions or by intentional design, has a way of signalling to operators or users when the technology requires modification to prevent failure or to enhance its performance.* | It's difficult to know if there are issues until the technology is already having major issues (e.g. toilets can't be flushed). Difficult to know if effluent is being treated adequately. | Remote flow sensing, or flow analogue gauge of flow rates through inlets, chambers and outlets would indicate if the ABR was failing. |
| **Fail-operational**  *The technology can still provide its overall function even when components or processes are damaged/compromised and undergoing repair.* | This depends on the degree of damage. It may provide a limited function if damaged (i.e. minor leakage of waste). However, some repairs may require inputs to be stopped or sludge to be removed to access areas for repair. It's not straightforward to isolate chambers. | Options to bypass chambers for repairs when needed may mean the ABR could continue to function while chambers were repaired. |
| **Decentralisation**  *Failures in decentralised systems (small-scale individual or clustered systems) are isolated locally which prevents failures from cascading and causing outages to many users/households.* | Compared to a septic tank, it is still possible that several households will have disrupted sanitation in the event of a failure. The assessment team noted there may be as many as 40 households connected to some ABRs. | Guidelines on maximum number of households to be connected to an ABR. |
| **Reusable materials**  *The materials from the destroyed technology can be reused for other purposes (including rebuilding the technology).* | The broken concrete is generally not easily used for other purposes. | If the chambers were built out of bricks, they could potentially be recovered and used for other purposes. |
| **Fail-silence**  *If the technology fails so that it cannot be used anymore, no failure will pose a continual health risk to the public or the environment beyond making the technology unavailable for use.* |  | Could consider HDPE linings around the outside of the ABR to limit public health risks in the event of leakage/damage. |
| **Accessibility for rapid flaw detection and repair**  *Components or processes of the technology can be easily accessed for examination.* | Access to some chambers may be difficult if full of sludge or water. This will also present a health risk to those doing repairs. There is also no way to shut off continued use during repairs. | Introduce shut off valve and ways to shut off toilets that feed into the ABR as needed. As noted earlier, the ability to bypass certain chambers would also help in repairing one chamber while others continue to function. A 'repair' procedure and required tools/equipment should be provided with each ABR, and ideally ABRs should only be installed where trained personnel are available to conduct repairs safely. |
| **Reciprocity**  *By providing its service, the technology produces outputs that build resilience in, or aid, another on-site or off-site system.* |  | If a soak pit or leach field was incorporated into the design, it could incorporate plantings to provide other functionality (e.g. animal feed), assuming it could be done without public health risk. |

Source: (Kohlitz et al., 2023)

# Supplementary material references

Kohlitz, J., Cunningham, I., & Willetts., J. (2023). *ClimateFIRST: Climate Framework to Improve the Resilience of Sanitation Technologies: How-to Guide*. Prepared by University of Technology Sydney, Institute for Sustainable Futures for the Bill and Melinda Gates Foundation.

Page, M. J., McKenzie, J. E., Bossuyt, P. M., Boutron, I., Hoffmann, T. C., Mulrow, C. D., Shamseer, L., Tetzlaff, J. M., Akl, E. A., Brennan, S. E., Chou, R., Glanville, J., Grimshaw, J. M., Hróbjartsson, A., Lalu, M. M., Li, T., Loder, E. W., Mayo-Wilson, E., McDonald, S., … Moher, D. (2021). The PRISMA 2020 statement: An updated guideline for reporting systematic reviews. *BMJ*, *372*, n71. https://doi.org/10.1136/bmj.n71

UTS-ISF. (2023). *ClimateFIRST outputs*. Institute for Sustainable Futures. https://www.uts.edu.au/isf/explore-research/projects/climate-resilient-urban-sanitation/climatefirst-outputs
